# Multiple variations in pulmonary veins during a thoracoscopic right lower lobectomy: A case report

**DOI:** 10.1111/1759-7714.13293

**Published:** 2020-01-28

**Authors:** Dario Amore, Dino Casazza, Umberto Caterino, Alessandro Saglia, Carlo Bergaminelli, Marcellino Cicalese, Pasquale Imitazione, Maria Rosaria Valentino, Roberta Civiletti, Carlo Curcio

**Affiliations:** ^1^ Department of Thoracic Surgery Monaldi Hospital Naples Italy; ^2^ Thoracic Endoscopic Unit Monaldi Hospital Naples Italy; ^3^ Department of Respiratory Diseases University of Naples Federico II, Monaldi Hospital Naples Italy

**Keywords:** Lung cancer, pulmonary veins, thoracoscopic surgical procedures

## Abstract

A knowledge of pulmonary vein anatomy variants allows an appropriate preoperative radiological assessment and safe surgical management of vascular anomalies in patients undergoing major lung resections. In our case, multiple pulmonary vein variations were identified pre‐ and intraoperatively in a patient undergoing thoracoscopic right lower lobectomy and included superior and common basal veins from the right lower lobe draining separately into the left atrium, middle lobe veins joining the superior segment right lower lobe vein and additional superior segment right lower lobe vein draining directly into the left atrium. The recognition of these anatomical abnormalities in pulmonary veins may help thoracic surgeons avoid surgical complications in patients undergoing anatomical lung resections.

## Introduction

The great variability in pulmonary venous anatomy has been the subject of several radiologic studies as pulmonary veins play a critical role in the pathophysiology of atrial fibrillation and their anatomical variants are related to a higher arrhythmogenic potential. This important clinical issue in recent decades has also enabled anatomists, through dissection of human fixed cadaveric lungs, to identify variants in the number and course of pulmonary veins with particular care such as the medial and the lateral middle lobe pulmonary veins that drain, either as a common trunk or independently, directly into the left atrium or into the inferior pulmonary vein.^1^, ^2^, ^3^ Interesting case reports and reviews concerning anomalies in pulmonary venous drainage have been moreover published in the current thoracic surgery literature in order to make major lung resections performed via less invasive approaches a safer procedure.^4^, ^5^ Here, we report the coexistence of multiple and rare pulmonary veins variants identified in an adult patient: each of them, if not recognized, could lead to intraoperative or early postoperative complications.

## Case report

**Figure 1:**
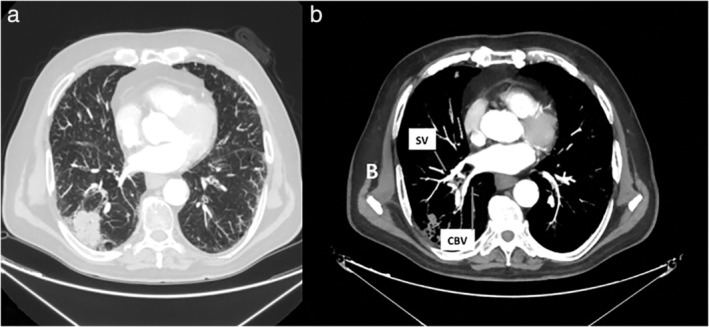
Chest CT scans with maximum intensity projection (MIP) reconstructions. (**a**) Peripheral solid lesion in the right lower lobe. (**b**) Superior and common basal veins from the right lower lobe draining separately into the left atrium. SV, superior vein; CBV, common basal vein.

**Figure 2:**
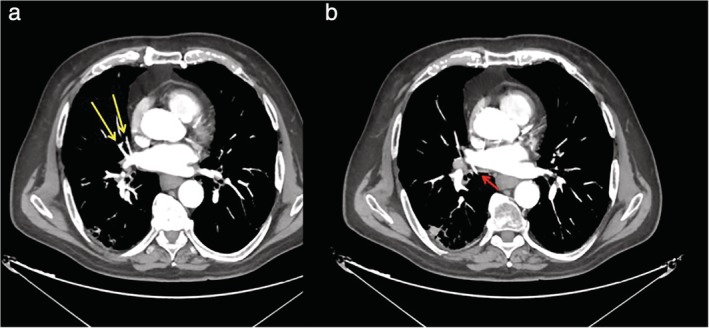
Contrast enhanced chest CT and MIP images in the assessment of venous pulmonary variants. (**a**) Middle lobe veins joining the superior segment right lower lobe vein (yellow arrows). (**b**) Independent connection of an additional superior segment right lower lobe vein direct to the left atrium (red arrow).

**Figure 3:**
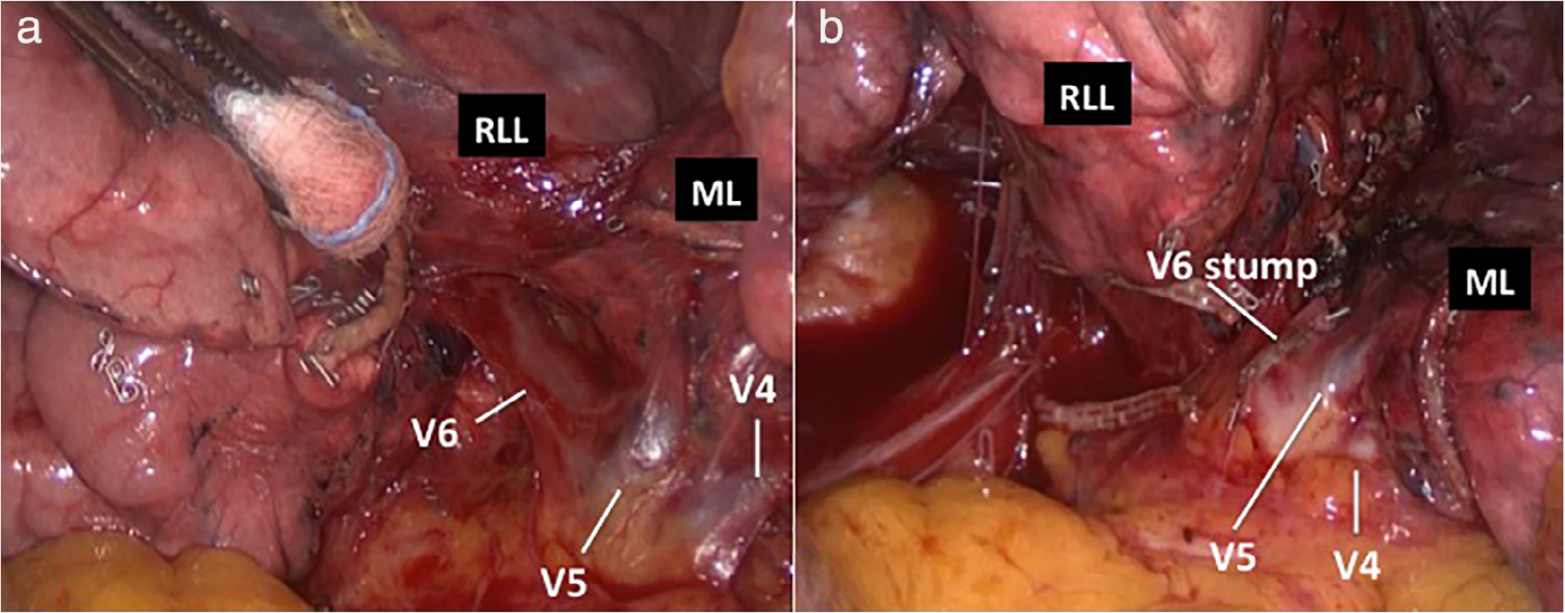
Intraoperative view of variations in draining patterns of right pulmonary veins at the hilum. (**a**) Veins (V4 and V5) draining blood from the right middle lobe into the superior segment right lower lobe vein (V6). (**b**) The stump of the divided vein draining the superior right lower lobe segment (V6) and preservation of the middle lobe veins (V4 and V5).

**Figure 4:**
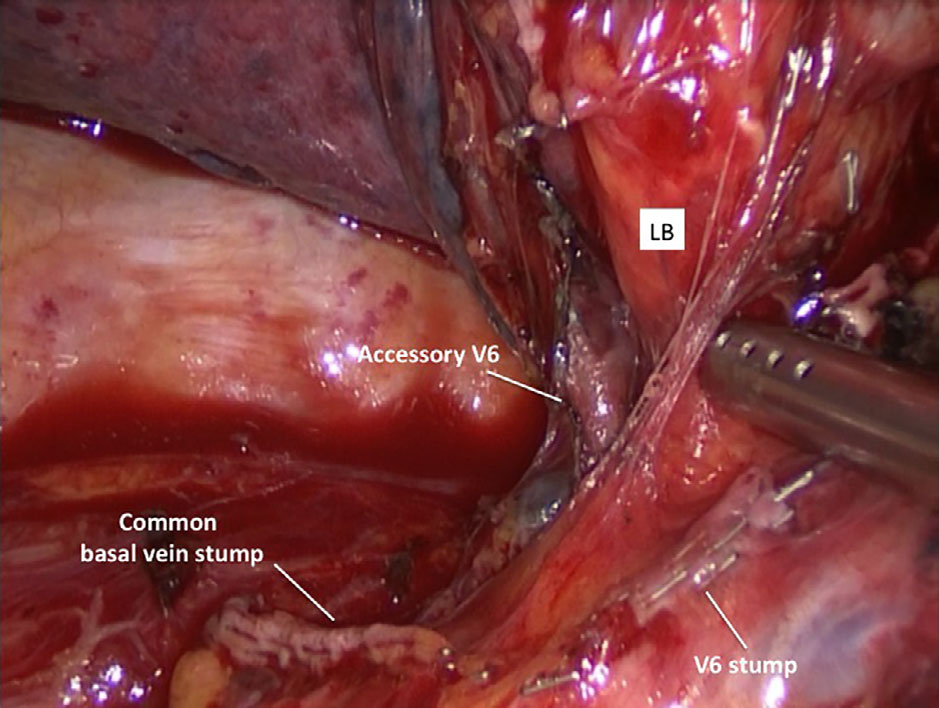
Accessory vein draining the superior right lower lobe segment (V6) located posterior to the right lower lobe bronchus. LB, lower bronchus.

A 79‐year‐old man was admitted to our operative unit for treatment of primary lung cancer located in the right lower lobe. He was classified as stage IIA, according to the eighth edition of the TNM staging system for lung cancer, and scheduled for video‐assisted thoracoscopic right lower lobectomy. Preoperative enhanced chest computed tomography (CT) scans showed a mass in the lower lobe of the right lung, measuring 48 × 32 mm and anatomical variations of pulmonary venous drainage including separate drainage of the superior segment right lower lobe vein (V6) from the basilar segments right lower lobe vein into the left atrium; middle lobe veins (V4 and V5) were found to empty into the V6 and accessory V6 passing behind the right lower lobe bronchus (Fig 1 and 2). During video‐assisted thoracoscopic surgery (VATS) lobectomy, after a careful hilar dissection and division of the anterior part of the major fissure, the inferior pulmonary vein was divided with a vascular stapler ensuring preservation of middle lobe venous drainage (Fig 3). Moreover, the correct identification of an accessory V6 during the posterior mediastinal lymph node dissection, allowed intraoperative bleeding to be avoided (Fig 4). The patient had an uneventful postoperative course and the pathological examination of the resected specimen resulted in a diagnosis of T2bN0M0 adenocarcinoma.

## Discussion

**Figure 5:**
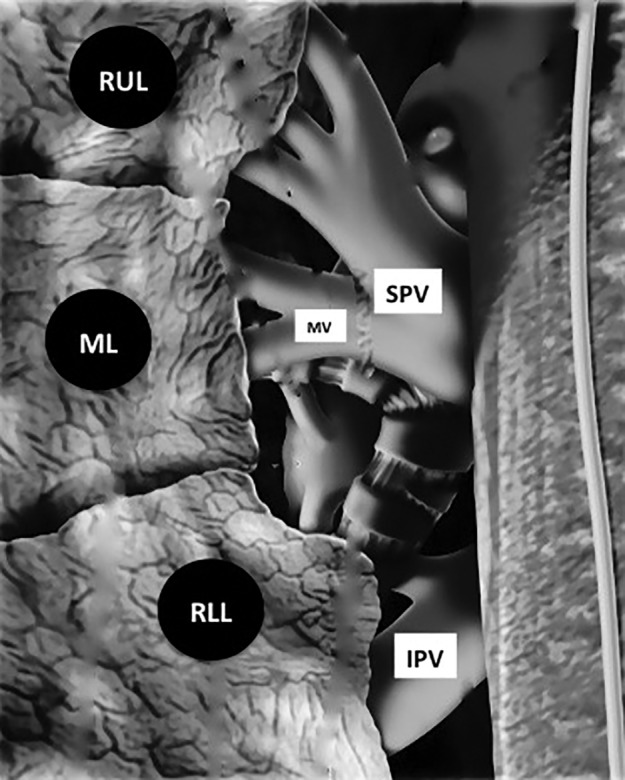
Two right pulmonary veins. The superior pulmonary vein drains the right upper and middle lobes; the inferior pulmonary vein drains the right lower lobe. IPV, inferior pulmonary vein; ML, middle lobe; MV, middle lobe veins; RLL, right lower lobe; RUL, right upper lobe; SPV, superior pulmonary vein.

Most individuals have four pulmonary veins in total; two on the left and two on the right with two separate ostia.^6^ (Fig 5). During the development of the human embryo, an initially solitary pulmonary vein is incorporated with its tributaries by an expanding left atrial wall: this progressive growth leads to four separate pulmonary veins with distinct ostia arising from the left atrium.^7^ The superior segment right lower lobe vein (V6) draining separately from the basilar segments right lower lobe vein into the left atrium, in a patient with a normal right upper lobe venous drainage, is a rare anatomical variant and requires a careful isolation of hilar structures to prevent the risk of intraoperative vessel injury. Variations in the number and course of pulmonary veins are more common on the right side and a great number of anomalies involve the venous drainage pattern of the right middle lobe.^2^, ^3^ In the case reported here, the veins draining the medial and the lateral segments of the middle lobe (V4 and V5) emptied separately into the superior segment right lower lobe vein (V6): this vascular anomaly has rarely been reported in the literature and preservation is essential during a right lower lobectomy to avoid severe pulmonary edema and potentially life‐threatening complications.^8^ Preoperatively and during lobar resection, we moreover identified an extra vein draining the superior right lower lobe segment, posterior to the right lower lobe bronchus and with independent atriopulmonary venous junction (accessory V6): if this anomalous vessel is not recognized during subcarinal lymph node dissection, there could be severe intraoperative bleeding due to massive blood flow from the left atrium. Lack of knowledge of the anatomical variations in the pulmonary venous system may reduce the possibility of their recognition with various imaging modalities. The added failure to identify abnormalities in pulmonary veins during major lung resections may consequently lead to surgical complications as intraoperative bleeding, severe pulmonary edema or modification and extension of the planned surgery.

## Disclosure

No authors do not report any conflict of interest.
